# Effectiveness of non-pharmacological therapies for chronic pain in people with autoimmune diseases in Africa: A protocol for a systematic review and meta-analysis

**DOI:** 10.1371/journal.pone.0306564

**Published:** 2024-07-02

**Authors:** Gebreamlak Gebremedhn Gebremeskel, Teklehaimanot Gereziher Haile, Gebremeskel Tukue Gebrewahd, Abrha Hailay, Woldu Aberhe, Guesh Mebrahtom, Kidane Zereabruk, Assefa Iyasu Negash, Hailemikael Gebrekidan, Degena Bahrey Tadesse

**Affiliations:** 1 Department of Adult Health Nursing, School of Nursing, College of Health Science, Aksum University, Aksum, Tigray, Ethiopia; 2 Department of Maternity and Neonatal Nursing, School of Nursing, College of Health Science, Aksum University, Aksum, Tigray, Ethiopia; 3 Department of Critical Care Nursing, School of Nursing, College of Health Science, Aksum University, Aksum, Tigray, Ethiopia; 4 Department of Child Health Nursing, School of Nursing, College of Health Science, Aksum University, Aksum, Tigray, Ethiopia; Universal Scientific Education and Research Network, UNITED KINGDOM

## Abstract

**Background:**

Autoimmune diseases affect 5–10% of the global population and cause chronic pain and impaired functionality. Chronic pain management involves pharmacological and non-pharmacological interventions, with non-pharmacological options gaining attention as safe, effective, and cost-effective alternatives. However, further research is needed to determine the effectiveness of these therapies in African patients with autoimmune diseases, as existing evidence varies.

**Methods:**

This review protocol has been registered in the International Prospective Register of Systematic Reviews (PROSPERO, CRD42023449896). Electronic databases (PubMed, Africa Index Medicus, Cochrane Library, CINAHL, PsycINFO, and Web of Science) will be used for searching published articles. The study will use R for data synthesis, employing a random-effects meta-analysis approach to calculate pooled effect sizes, assess heterogeneity using the I^2^ statistic, and evaluate publication bias. In conclusion, this protocol aims to fill the knowledge gap on non-pharmacological therapies for chronic pain in patients with autoimmune diseases in Africa. It will potentially enhance evidence-based decision-making to improve pain management and, hence, the quality of life of people with autoimmune diseases in Africa.

## Introduction

The availability of population-based data on autoimmune diseases (ADs) in Africa is limited. However, ADs affect approximately 5–10% of the global population [1, 2], and their prevalence is on the rise, leading to significant health and economic challenges for individuals and society [3]. ADs are chronic conditions characterized by inflammation, tissue damage, and a wide range of symptoms. This inflammation results from a breakdown of immune tolerance, leading the immune system to attack healthy tissues [4]. Among individuals with established ADs such as rheumatoid arthritis and lupus, chronic pain significantly affects most patients, potentially leading to impaired functionality, physical well-being, and reduced quality of life. Accordingly, the management of chronic pain is of paramount importance for people with ADs [5–7].

Both pharmacological and non-pharmacological interventions are used to manage chronic pain in ADs, and pharmacological treatments typically have a wide range of adverse effects because they are nondisease-specific and have broad action [8]. Non-pharmacological interventions include physical activity, lifestyle interventions, mindfulness-based interventions, and potentially complementary and alternative medicines. Complementary and alternative medicines could be important in the treatment of chronic pain in people with ADs in Africa because, according to a systematic review, 58.2% of people in sub-Saharan Africa use traditional and complementary alternative medicine for their health problems [9].

While the majority of research on non-pharmacological pain management in ADs has been conducted outside Africa, there is a need for further investigation into its effectiveness among patients with ADs in Africa, as existing evidence varies. Therefore, conducting a systematic review and meta-analysis can help address these discrepancies by pooling data and providing more reliable and comprehensive results on the effectiveness of non-pharmacological therapies for managing chronic pain in people with ADs in Africa. Additionally, this review will inform clinical practice, policymaking, and future research endeavors and ultimately improve the quality of life and health outcomes of patients with ADs in Africa.

## Methods

### Design

This protocol was reported according to the Preferred Reporting Items for Systematic Review and Meta-analysis Protocols (PRISMA-P) and registered in PROSPERO (CRD42023449896).

### Search strategies

To identify relevant studies assessing the effectiveness of non-pharmacological therapies for chronic pain in people with ADs, a comprehensive search will be conducted across various important electronic databases. These electronic databases will be PubMed, Africa Index Medicus, the Cochrane Library, CINAHL, PsycINFO, and the Web of Science [10]. The search strategy will employ a combination of Medical Subject Headings (MeSH) terms for PubMed and appropriate indexing terms and keywords for other databases, utilizing appropriate Boolean operators (AND, OR). The search strategy will encompass all available literatures published from 2020 until the most up-to-date evidence at the time of manuscript submission. The search strategy will include relevant terms pertaining to four categories: 1) "ADs," 2) "chronic pain," 3) "non-pharmacological therapies," and 4) "Africa." The search terms are explained in S1 Table.

### Eligibility criteria

#### Inclusion criteria

➢ Population: All patients diagnosed with an ADs
➢ Intervention: Studies with non-pharmacological therapies
➢ Comparator: People with ADs without chronic pain in Africa
➢ Outcome: Studies that reported the effectiveness of non-pharmacological therapies for chronic pain
➢ Study designs: Randomized controlled trials, cohort studies, and qualitative studies
➢ Study setting: Studies conducted in Africa
➢ Language: Studies published in English

#### Exclusion criteria

➢ Study designs (protocol articles, reviews articles, case reports, case series, cross-sectional studies, and case-control studies).
➢ Duplicate studies.

### Data management and extraction

Two independent reviewers will extract data from the included studies using a standardized data extraction form. The extracted data will include study characteristics (e.g, author, year, country, sample size), participant characteristics (e.g., age, gender, AD type), intervention details (e.g., type, frequency, duration), outcomes, and adverse events.

More specifically, we will extract the following information: publication, authors, month, country, and type of publication. Additionally, study characteristics such as the design, setting, sample size, response rate, mean or median age, or age range of participants will be recorded. Outcomes of interest, including pain intensity, pain-related disability, quality of life, and adverse events, will also be extracted, along with information on the burden of chronic pain and its impact on patients’ lives. Outcome measures of interest will be pain intensity and pain interference. The secondary outcomes will be quality of life and adverse events.

### Assessment of quality of evidence and risk of bias

The Newcastle-Ottawa quality assessment tool will be used to assess the methodological quality of cohort studies in this review [11]. In addition, we will utilize the Cochrane risk-of-bias tool and Critical Appraisal Skills Program (CASP) to assess the methodological quality of RCTs and qualitative studies respectively [12, 13].

Two independent reviewers will assess the risk of bias in the included studies using the Cochrane risk of bias tool. The tool assesses the risk of bias in the following domains: random sequence generation, allocation concealment, blinding of participants and personnel, blinding of outcome assessment, incomplete outcome data, selective reporting, and other sources of bias. Any disagreements will be resolved through discussion or consultation with a third reviewer.

### Data synthesis and analysis

The data will be synthesized using a random-effects model meta-analysis. Heterogeneity will be assessed using the I^2^ statistic and the chi-squared test. Subgroup, sensitivity, and influence analysis (exploring possible effects of outliers) will be conducted to explore the potential sources of heterogeneity and to assess the robustness of the findings whenever necessary.

The data analysis will be conducted using R software, with the Metafor package utilized for calculating the standardized mean difference (SMD) with 95% confidence intervals (CIs) for continuous outcomes and the risk ratio (RR) with 95% CI for dichotomous outcomes. The resulting data will be presented through graphical and tabular summaries, such as forest plots and summary tables.

We will conduct subgroup analysis based on the type of non-pharmacological therapy, the type of AD, the duration of therapy, and the quality of the included studies. We will conduct a sensitivity analysis to assess the impact of removing studies with a high risk of bias or studies with a small sample size on the overall effect size. Publication bias will be assessed using Egger’s test and visual inspection of the funnel plots whenever possible.

## Discussion

The aim of this systematic review and meta-analysis will be to assess the effectiveness of non-pharmacological therapies for managing chronic pain in patients with ADs in Africa. The overarching goal is to fill this knowledge gap and provide evidence-based insights in a context where research on this topic is limited. This review will analyze the available literature, including studies on interventions such as exercise, physical therapy, cognitive-behavioral therapy, mindfulness, meditation, mind-body therapies, traditional African herbal remedies, and self-management strategies. The systematic review will be published in accordance with the PRISMA guidelines [14].

Non-pharmacological therapies are essential for pain management in Africa due to their cost-effectiveness, cultural relevance, and minimal adverse effects. Exploring the effectiveness of traditional African medicines in managing chronic pain associated with ADs holds promise for a more inclusive and culturally sensitive healthcare approach in Africa. Patient perspectives on traditional remedies are increasingly important, and some plants, as evidenced by Khumalo GP et. al. (2022), have already shown promise in managing inflammatory pain [15]. However, robust research and collaboration are crucial to ensuring their safe and effective integration alongside existing therapies.

Potential setbacks include the scarcity of relevant studies and the heterogeneity of interventions and outcome measures. To overcome these challenges, a comprehensive search strategy will be employed, and experts in the field will be consulted. Despite these setbacks, this review is expected to yield valuable insights for healthcare professionals and identify research gaps, thus improving pain management and quality of life for patients with ADs in Africa.

## Conclusion

In conclusion, this systematic review and meta-analysis will aim to fill the knowledge gap regarding the effectiveness of non-pharmacological therapies for managing chronic pain in people with ADs in Africa. The findings from this study will provide valuable insights into the potential benefits of interventions such as exercise, physical therapy, cognitive-behavioral therapy, mindfulness, and meditation. These insights will inform evidence-based decision-making, improve pain management strategies, and enhance the quality of care and quality of life for individuals in this population. Furthermore, the comprehensive synthesis of the available evidence may also inform clinical practice and future research in this field.

## Supporting information

S1 Checklist PRISMA 2020 checklist. (DOCX)

S1 Table Search strategy. (DOCX)
