# Supplementary material for: Effectiveness of non-pharmacological therapies for chronic pain in people with autoimmune diseases in Africa: A protocol for a systematic review and meta-analysis
Source: PLoS One. 2024 Jul 2;19(7):e0306564. doi: 10.1371/journal.pone.0306564 (PMC11218934; doi:10.1371/journal.pone.0306564)
Supplement: S1 Table — (DOCX) [file pone.0306564.s002.docx]

**Supplementary Table 1: Search strategy**

| ***Databases*** | ***Search terms*** |
| --- | --- |
| PubMed | *("****Autoimmune diseases****"* *[Mesh] OR "Autoimmune disease” [Mesh] OR “Rheumatoid arthritis" [Mesh] OR “Rheumatoid” [Mesh] OR "Systemic lupus erythematosus" [Mesh] OR “Lupus” [Mesh] OR "Multiple sclerosis" [Mesh] OR "Ankylosing Spondylitis"* *[Mesh] OR "Psoriasis" [Mesh] OR "Hashimoto's thyroiditis" [Mesh] OR "Graves' disease" [Mesh] OR "Celiac disease" [Mesh] OR "Inflammatory bowel disease" [Mesh] OR "Crohn's disease" [Mesh] OR* ***"Inflammatory Diseases" [Mesh]* OR** *"ulcerative colitis" [Mesh] OR "Sjögren's syndrome" [Mesh] OR "Scleroderma" [Mesh] OR "Myasthenia gravis" [Mesh] OR "Autoimmune hepatitis" [Mesh] OR "Sydenham's chorea" [Mesh])* ***AND***  ***#2****("****Pain****" [Mesh] OR "painful" [Mesh] OR "discomfort" [Mesh] OR "****aching****"* *[Mesh]* ***OR*** *"****stiffness****"* *[Mesh]* ***OR*** *"****tenderness****"* *[Mesh]* ***OR*** *"****chronic pain****"* *[Mesh]* ***OR*** *"****persistent pain****"* *[Mesh]* ***OR*** *"****long-term pain****" [Mesh])* ***AND***  ("**Non-Pharmacological Therapies**" *[Mesh]* OR *"*non-pharmacological intervention*"* *[Mesh]* OR *"*non-pharmacological treatment*"* *[Mesh]* ***OR*** *"Complementary* ***therapy****"* *[Mesh]* ***OR*** *"****alternative therapy****"* *[Mesh]* ***OR*** *"Therapeutics" [Mesh] OR "Therapeutic"* *[Mesh]* ***OR*** *"****exercise****"* *[Mesh]* ***OR*** *"****physical therapy****"* *[Mesh]* ***OR*** *"****cognitive-behavioral therapy****"* *[Mesh]* ***OR*** *"****mindfulness****"* *[Mesh]* ***OR*** *"****meditation*** *" [Mesh]* ***OR*** *"****Psychological therapy****"* *[Mesh]* ***OR*** *"****Movement therapy****"* *[Mesh]* ***OR*** *"****Nutritional therapy****"* *[Mesh]* ***OR*** *"****life style interventions****"* *[Mesh]* ***OR*** *"****massage therapy****"* *[Mesh]* ***OR*** *"****mind-body therapies****"* *[Mesh]* ***OR*** *"traditional African herbal remedies"* *[Mesh]* ***OR*** *"****self-management strategies****" [Mesh])* ***AND***  ***(****"****Africa****"* *[Mesh] OR "Angola" [Mesh] OR "Benin" [Mesh] OR "Botswana" [Mesh] OR "Burkina Faso" [Mesh] OR "Burundi" [Mesh] OR "Cabo Verde" [Mesh] OR "Cameroon" [Mesh] OR "Canary Islands" [Mesh] OR "Central African Republic" [Mesh] OR "Chad" [Mesh] OR "Comoros" [Mesh] OR "Congo" [Mesh] OR "Democratic Republic of the Congo" [Mesh] OR "Djibouti" [Mesh] OR "Equatorial Guinea" [Mesh] OR "Eritrea" [Mesh] OR "Eswatini" [Mesh] OR "Ethiopia" [Mesh] OR "Gabon" [Mesh] OR "Gambia" [Mesh] OR "Ghana" [Mesh] OR "Guinea" [Mesh] OR "Guinea Bissau" [Mesh] OR "Ivory Coast" [Mesh] OR "Cote d’Ivoire" [Mesh] OR "Kenya" [Mesh] OR "Lesotho" [Mesh] OR "Liberia" [Mesh] OR "Madagascar" [Mesh] OR "Malawi" [Mesh] OR "Mali" [Mesh] OR "Mauritania" [Mesh] OR "Mauritius" [Mesh] OR "Mayotte" [Mesh] OR "Mozambique" [Mesh] OR "Namibia" [Mesh] OR "Niger" [Mesh] OR "Nigeria" [Mesh] OR "Réunion" [Mesh] OR "Rwanda" [Mesh] OR "Sao Tome and Principe" [Mesh] OR "Senegal" [Mesh] OR "Seychelles" [Mesh] OR "Sierra Leone" [Mesh] OR "Somalia" [Mesh] OR "South Africa" [Mesh] OR “South Sudan” [Mesh] OR "Saint Helena" [Mesh] OR "Togo" [Mesh] OR "Uganda" [Mesh] OR “United Republic of Tanzania” [Mesh] OR "Zambia" [Mesh] OR "Zimbabwe" [Mesh] OR "Sub-Saharan Africa" [Mesh] OR "Sub-Saharan African" [Mesh] OR "Sub-Saharan Africa" [Mesh] OR "Sub-Saharan African" [Mesh] OR "Eastern Africa" [Mesh] OR "West Africa" [Mesh] OR "West African" [Mesh] OR "Western African" [Mesh] OR “Middle Africa” [Mesh] OR "Southern Africa" [Mesh] OR "Western Africa" [Mesh] OR "Western Sahara" [Mesh] OR "East Africa" [Mesh] OR "East African" [Mesh] OR "Eastern Africa" [Mesh] OR "Central Africa" [Mesh] OR "Central African" [Mesh] OR "South African" [Mesh] OR "Southern African" [Mesh])* |
| ***Other databases (***such as, Africa Index Medicus, Cochrane Library, CINAHL, PsycINFO, and Web of Science***)*** | *("****Autoimmune diseases****" OR “Autoimmune disease” OR “Rheumatoid arthritis" OR “Rheumatoid” OR "Systemic lupus erythematosus" OR “Lupus” OR "Multiple sclerosis" OR "Ankylosing Spondylitis" OR "Psoriasis" OR "Hashimoto's thyroiditis" OR "Graves' disease" OR "Celiac disease" OR "Inflammatory bowel disease" OR "Crohn's disease" OR "Inflammatory Diseases" OR "ulcerative colitis" OR "Sjögren's syndrome" OR "Scleroderma" OR "Myasthenia gravis" OR "Autoimmune hepatitis" OR "Sydenham's chorea")* ***AND***  *("****Pain****" OR "painful" OR "discomfort" OR "aching" OR "stiffness" OR "tenderness" OR "chronic pain" OR "persistent pain" OR "long-term pain")* ***AND***  *("****Non-Pharmacological Therapies****" OR "non-pharmacological intervention" OR " non-pharmacological treatment" OR "Complementary therapy" OR "alternative therapy" OR "Therapeutics" OR " Therapeutic" OR "exercise" OR "physical therapy" OR "cognitive-behavioral therapy" OR "mindfulness" OR "meditation" OR "Psychological therapy" OR "Movement therapy" OR "Nutritional therapy" OR "life style interventions" OR "massage therapy" OR "mind-body therapies" OR "traditional African herbal remedies" OR "self-management strategies")* ***AND***  ***(****"* ***Africa****" OR "Angola" OR "Benin" OR "Botswana" OR "Burkina Faso" OR "Burundi" OR "Cabo Verde" OR "Cameroon" OR "Canary Islands" OR "Central African Republic" OR "Chad" OR "Comoros" OR "Congo" OR "Democratic Republic of the Congo" OR "Djibouti" OR "Equatorial Guinea" OR "Eritrea" OR "Eswatini" OR "Ethiopia" OR "Gabon" OR "Gambia" OR "Ghana" OR "Guinea" OR "Guinea Bissau" OR "Ivory Coast" OR "Cote d’Ivoire" OR "Kenya" OR "Lesotho" OR "Liberia" OR "Madagascar" OR "Malawi" OR "Mali" OR "Mauritania" OR "Mauritius" OR "Mayotte" OR "Mozambique" OR "Namibia" OR "Niger" OR "Nigeria" OR "Réunion" OR "Rwanda" OR "Sao Tome and Principe" OR "Senegal" OR "Seychelles" OR "Sierra Leone" OR "Somalia" OR "South Africa" OR “South Sudan” OR "Saint Helena" OR "Togo" OR "Uganda" OR “United Republic of Tanzania” OR "Zambia" OR "Zimbabwe" OR "Sub-Saharan Africa" OR "Sub-Saharan African" OR "Sub-Saharan Africa" OR "Sub-Saharan African" OR "Eastern Africa" OR "West Africa" OR "West African" OR "Western African" OR “Middle Africa” OR "Southern Africa" OR "Western Africa" OR "Western Sahara" OR "East Africa" OR "East African" OR "Eastern Africa" OR "Central Africa" OR "Central African" OR "South African" OR "Southern African")* |
